# Pressure‐Derived Right Ventricular Ejection Fraction During Initial Dual Combination Therapy in Pulmonary Arterial Hypertension

**DOI:** 10.1002/pul2.70394

**Published:** 2026-09-03

**Authors:** Tharsiga Kanagasingam, Jonas Lund, Christoph R. Sinning, Gunnar K. Lund, Hans Klose, Lars Harbaum

**Affiliations:** ^1^ Division of Respiratory Medicine, Department of Internal Medicine II University Medical Centre Hamburg‐Eppendorf Hamburg Germany; ^2^ Department of Diagnostic and Interventional Radiology University Medical Centre Hamburg‐Eppendorf Hamburg Germany; ^3^ Department of Cardiology, University Heart and Vascular Centre Hamburg University Medical Centre Hamburg‐Eppendorf, Member of the German Centre for Cardiovascular Research (DZHK), Partner Site Hamburg/Kiel/Luebeck Hamburg Germany

## Abstract

In patients with pulmonary arterial hypertension, pressure‐based right ventricular ejection fraction (RVEF) estimated from routine right heart catheterisation correlated with RVEF from magnetic resonance imaging. A framework combining Weibull distribution‐derived theoretical isovolumic pressure and time‐varying elastance‐derived end‐systolic pressure showed the closest correlation and was responsive to initial oral combination therapy.

## 1 Introduction

The primary determinant of outcomes in pulmonary arterial hypertension (PAH) is the function of the right ventricle (RV). Reducing afterload through PAH‐targeted therapy is associated with improved RV function [1]. Recently, a pressure‐derived framework has been proposed to quantify RV ejection fraction (RVEF) from pressure–time curves recorded during right heart catheterisation (RHC). This pressure‐derived RV framework relies on two key pressure measurements: the theoretical maximal RV pressure estimated under the assumption of isovolumic contraction (Piso) and the end‐systolic pressure (ESP). These measures can be combined to approximate RVEF as (Piso—ESP)/Piso, or equivalently 1—(ESP/Piso) [2]. Thus, pressure‐derived RVEF can be conceptualised as the fraction of Piso not occupied by ESP. We sought to evaluate the performance of pressure‐derived RVEF against cardiac magnetic resonance imaging (MRI)‐derived RVEF in PAH and to assess its response to first‐line combination therapy with phosphodiesterase type 5 inhibitors (PDE5I) and endothelin receptor antagonists (ERA).

## 2 Methods

Two independent cohorts of patients with PAH from the same centre were included in this study. The first prospective cohort comprised patients who underwent cardiac MRI and RHC within 24 h. The second retrospective cohort comprised patients who underwent RHC both at baseline and within 24 months after initiation of first‐line dual oral combination therapy (PDE5I and ERA). Patients attended the University Medical Centre Hamburg‐Eppendorf, Germany, between 2021 and 2024. PAH was defined by a mean pulmonary arterial pressure (mPAP) > 20 mmHg, a pulmonary arterial wedge pressure (PAWP) ≤ 15 mmHg, and a pulmonary vascular resistance (PVR) ≥ 3 Wood units and classified according to current international guidelines [3].

RV pressure–time curves were obtained from standard fluid‐filled catheter systems. Piso was estimated using three methods: a four‐parameter Weibull model, a sinusoidal function, and the intersection of two tangential lines [2, 4, 5]. ESP was estimated using four different approaches: time‐varying elastance‐derived ESP (ESPtve) from the squared second derivative of the RV pressure signal, mPAP, adjusted mPAP (1.65 x mPAP—7.79), and mean pulmonary artery diastolic pressure (PADP) [2, 4, 5, 6]. Pressure–time recordings were manually segmented and visually inspected after automated beat‐by‐beat calculation of RV pressure‐derived metrics. Values were subsequently averaged across the entire recording. In addition to pressure‐derived RVEF, we calculated effective arterial elastance (Ea) and end‐systolic elastance (Ees) using the pressure‐only single‐beat framework. Stroke volume (SV) was derived from catheter‐based cardiac output, and Ea and Ees were calculated as ESP/SV and (Piso−ESP)/SV, respectively. These formulations are independent of assumptions regarding the volume‐axis intercept (V0). Furthermore, statistical analyses included Bland–Altman analysis, Wilcoxon matched‐pairs signed‐rank testing, receiver operating characteristic (ROC) analysis, and Spearman's rank correlation. Data are presented as mean ± standard deviation.

## 3 Results

**Figure 1:**
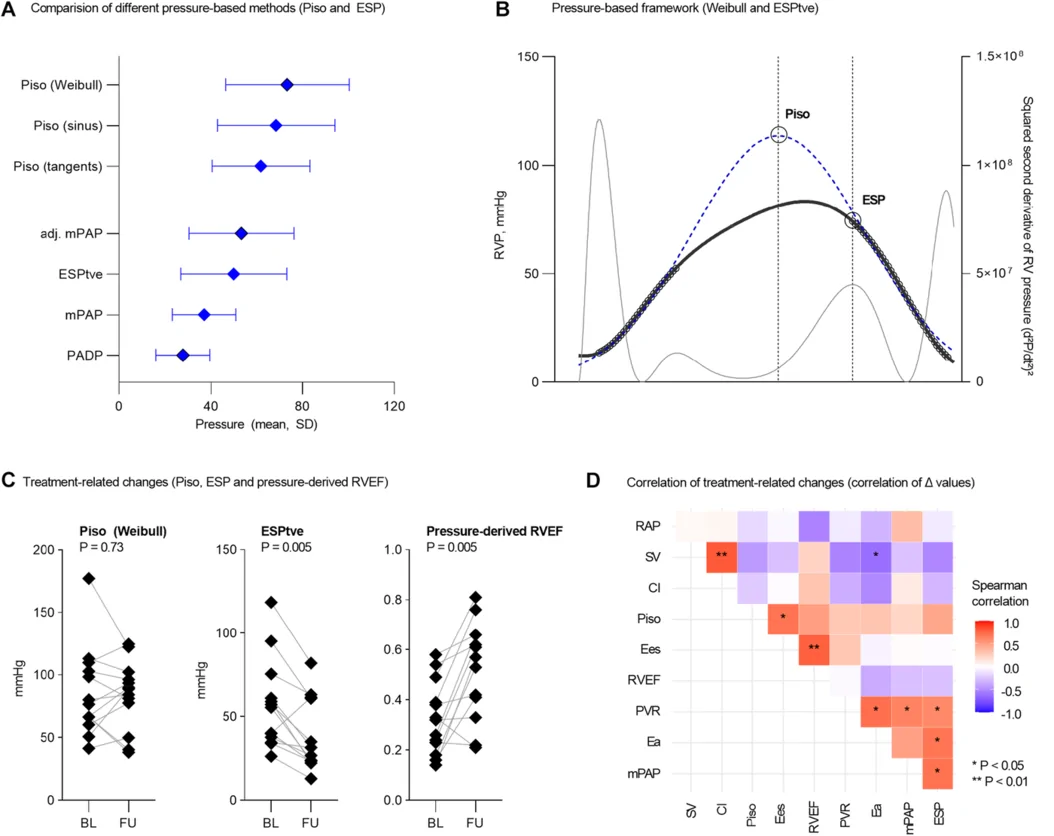
Pressure‐derived estimation of RVEF and response to initial dual PAH‐targeted therapy. (A) Comparison of different pressure‐derived methods to estimate maximal isovolumentric pressure (Piso) and end‐systolic pressure (ESP), with mean and standard deviation (SD) provided. (B) Right ventricular (RV) pressure–time curve demonstrating estimation of Piso using a four‐parametric Weibull distribution function (blue dashed line) and ESP using the time‐varying elastance approach (ESPtve) based on the squared second derivative of the pressure signal (grey line). Pressure‐derived RV ejection fraction (RVEF) was calculated as 1—(ESP/Piso). (C) Treatment‐related changes in pressure‐derived RV metrics in 13 incident PAH patients receiving first‐line dual oral combination therapy with tadalafil and macitentan (*p*‐values obtained from Wilcoxon matched‐pairs signed‐rank testing). (D) Correlation matrix based on treatment‐related changes (correlation of Δ values) from right heart catheterisation at baseline and following treatment initiation. End‐systolic elastance (Ees) was calculated as (Piso‐ESP)/SV and effective arterial elastance (Ea) as ESP/SV. BL, baseline; CI, cardiac index; FU, follow‐up; PADP, pulmonary arterial diastolic pressure; mPAP, mean pulmonary arterial pressure; MRI, magnetic resonance imaging; PAH, pulmonary arterial hypertension; PVR, pulmonary vascular resistance; RAP, right atrial pressure; SV, stroke volume.

In the first cohort of 36 prevalent PAH patients, the mean age was 54 ± 12 years, 69% were female, and 61% had idiopathic or heritable PAH. Comparison of the different methods used to estimate the components of pressure‐derived RVEF demonstrated similar Piso values across methods, with no statistically significant differences (the comparison between Weibull and tangent extrapolation approached significance, *p* = 0.08; Figure 1A). In contrast, greater variation was observed among the ESP surrogates. ESPtve and adjusted mPAP yielded comparable values with no significant differences, whereas conventional mPAP and PADP provided systematically lower estimates.

We generated pressure‐derived RVEF based on all possible 12 combinations. The closest correlation of MRI‐derived RVEF was obtained using the combination of a Weibull‐derived Piso and ESPtve (*r* = 0.68, 95% CI 0.43 to 0.83; Figure 1B) [2]. Pressure‐derived RVEF ranged from 7% to 68% (mean 34% ± 17%), whereas MRI‐derived RVEF ranged from 12% to 68% (42% ± 15%). Pressure‐derived RVEF demonstrated a mean bias of 7%, with limits of agreement ranging from −14% to 28%. Because an MRI‐derived RVEF ≤ 35% has been associated with adverse outcomes, we assessed the ability of pressure‐derived RVEF to identify patients below this threshold. The area under the ROC curve was 0.83 (95% confidence interval [CI], 0.70–0.96), confirming previous observations [7]. A pressure‐derived RVEF cut‐off of 41% achieved 100% sensitivity.

In the second cohort of 13 incident PAH patients, we evaluated treatment‐related changes within the pressure‐derived RV framework. The mean age was 63 ± 10 years, 60% were female, and 73% had idiopathic or heritable PAH. All patients initiated dual oral combination therapy with tadalafil 40 mg and macitentan 10 mg once daily. Follow‐up RHC was performed after a mean of 9.7 ± 6.1 months. Treatment decreased ESPtve from 56 ± 27 to 38 ± 21 mmHg, whereas Piso (Weibull) did not significantly change after treatment initiation (Figure 1C). Pressure‐derived RVEF increased from 33 ± 14 to 52% ± 19% (Figure 1C). Applying the pressure‐derived RVEF cut‐off of 41%, 10 of 13 patients (77%) were classified below the threshold before treatment compared with only 3 of 13 patients (23%) after treatment initiation (Fisher's exact test, *p* = 0.017). Treatment‐associated changes in ESPtve were positively correlated with changes in mPAP, PVR and Ea, consistent with ESP reflecting pulmonary vascular load. Whereas changes in Piso (Weibull) correlated positively with changes in Ees (Figure 1D).

## 4 Discussion

The present study demonstrates that pressure‐derived metrics obtained from the single‐beat framework provide a physiologically coherent assessment of RV function in PAH. Various approaches have been proposed to estimate Piso and ESP from pressure–time curves. Among the evaluated methods, the combination of a Weibull‐derived Piso and time‐varying elastance‐derived ESP showed the closest correlation with MRI‐derived RVEF in our cohort and accurately identified patients with prognostically relevant RV dysfunction. In line with prior studies, Piso estimated using a Weibull distribution yielded results comparable to conventional sinusoidal extrapolation methods [2]. Likewise, validation against pressure–volume loop measurements has demonstrated that ESP derived from maximal time‐varying elastance closely approximates directly measured end‐systolic pressure, whereas the use of mPAP as a surrogate is less accurate [8].

Pressure‐derived RVEF increased following initiation of dual oral combination therapy. The magnitude of improvement was consistent with changes in MRI‐derived RVEF reported in the REPAIR study, in which treatment with macitentan, with or without a PDE5 inhibitor, increased RVEF by approximately 10% after 52 weeks [9]. The observed treatment response within the pressure‐derived framework was driven by a reduction in ESP, whereas Piso remained largly unchanged. Because ESP correlated with changes in mPAP and Ea during treatment for PAH, the observed increase in pressure‐derived RVEF appears to have been driven predominantly by unloading of the RV. Whether pressure‐derived RVEF provides clinically meaningful information beyond conventional haemodynamic measures of pulmonary vascular load therefore remains uncertain. Nevertheless, pressure‐derived RVEF integrates ESP with the theoretical pressure reserve represented by Piso and therefore may provide a more physiologically intuitive measure of RV systolic function than pulmonary pressure alone.

The pressure‐only single‐beat framework may provide additional physiological insight [10]. When combined with SV, Ees and Ea can be estimated. Notably, the ratio Ees/Ea, a measure of RV–pulmonary arterial coupling, simplifies to (Piso—ESP)/ESP because SV cancels out. Thus, both pressure‐derived RVEF and Ees/Ea are determined solely by the relationship between Piso and ESP and can be derived without volumetric calibration. Indeed, pressure‐derived RVEF is mathematically linked to RV–pulmonary arterial coupling according to RVEF = (Ees/Ea)/(1 + Ees/Ea). Based on this relation, the pressure‐derived RVEF threshold of 41% identified in our study corresponds to an Ees/Ea ratio of approximately 0.7, a value that has been interpreted as the transition from relatively preserved to impaired RV–pulmonary arterial coupling, with prognostic implication in PAH [11, 12].

However, interpretation of these pressure‐derived estimates as absolute measures of ventricular contractility remains limited when compared with gold‐standard pressure–volume loop analyses. In patients with PAH, V0 may differ from zero and may change with RV remodelling or in response to therapy, potentially affecting the relationship between pressure‐derived indices and true pressure–volume loop‐derived ventricular mechanics [13, 14].

Additional limitations of our study merit consideration. Our single‐beat framework relied on idealised curve fitting and manual segmentation of pressure recordings, both of which may introduce measurement error. More recently, Piso estimation based on the intersection of tangents through inflection points of the RV pressure waveform has been proposed as a computationally simpler alternative for continuous implementation [5]. Although this approach may support automation, it showed lower agreement with MRI‐derived RVEF in our cohort. Finally, our analyses were performed in relatively small cohorts from a single centre and RV volumetric data were not available in the longitudinal cohort precluding direct comparison between pressure‐ and volume‐derived measures of RV function.

## 5 Conclusions

Our findings contribute to the ongoing evaluation of pressure‐derived surrogates of RV function in PAH. Pressure‐derived RVEF could be estimated from routine RHC recordings, showed moderate correlation with MRI‐derived RVEF, and changed following initiation of PAH‐targeted therapy. However, the observed treatment response appeared to be driven primarily by changes in afterload. Further studies are needed to clarify the clinical significance of pressure‐derived RVEF and to determine whether it provides information beyond conventional haemodynamic assessment of the pulmonary vasculature.

## Author Contributions

T.K., J.L., and L.H. conceived the study, analysed the data, and drafted the manuscript. All authors contributed to data acquisition, interpretation of the results, critical revision of the manuscript, and approval of the final version.

## Funding

The authors have nothing to report.

## Ethics Statement

All patients provided written informed consent (ClinicalTrials.gov identifier: NCT04654650). Local ethics committee approved the study (Ethik‐Kommission der Ärztekammer Hamburg, 2020‐10122_1‐BO‐ff).

## Conflicts of Interest

The authors declare no conflicts of interest.

## Guarantor

L.H. acts as guarantor of the work.

## Data Availability

The data that support the findings of this study are available from the corresponding author upon reasonable request.
